# P-1984. AB2CO: a mortality risk score for COVID-19 patients admitted to intensive care units

**DOI:** 10.1093/ofid/ofae631.2142

**Published:** 2025-01-29

**Authors:** Virginia Mara Reis Gomes, Magda Carvalho Pires, Polianna Delfino-Pereira, Vandack Nobre, Milena S Marcolino

**Affiliations:** Universidade Federal de Minas Gerais (UFMG), Belo Horizonte, Minas Gerais, Brazil; Universidade Federal de Minas Gerais (UFMG), Belo Horizonte, Minas Gerais, Brazil; Universidade Federal de Minas Gerais (UFMG), Belo Horizonte, Minas Gerais, Brazil; Universidade Federal de Minas Gerais (UFMG), Belo Horizonte, Minas Gerais, Brazil; Medical School, Universidade Federal de Minas Gerais, Belo Horizonte, Minas Gerais, Brazil

## Abstract

**Background:**

During the COVID-19 pandemic, several scoring systems have been developed and tested to stratify patients according to their risk levels, enabling the proper allocation of resources and increasing the likelihood of successful outcomes. However, there remains a gap in the availability of an effective tool for predicting in-hospital mortality among COVID-19 patients admitted to the intensive care unit (ICU). Most of the studies are bounded my methodological flaws which may lead to different bias, and uses data before the emergence of new variants and the broad use of vaccionation, that my impact scores' performance. We aimed to address this gap by developing a novel risk score and comparing it with other existing scores using more recent data.

Area under the ROC curve of the AB2CO risk score

**Figure:**
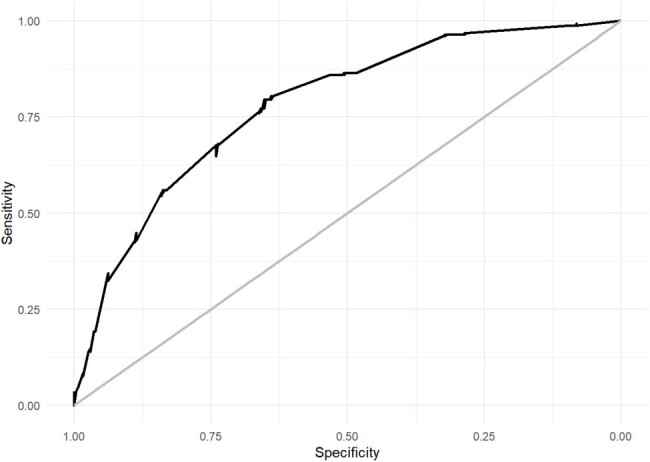

**Methods:**

This multicenter observational study included adult (≥18 years) patients with laboratory-confirmed COVID-19 who were admitted to 18 ICUs from 9 Brazilian cities, from September/2021 to July/2022. A total of 558 patients were included in the analysis, median age was 69 years (interquartile range 58-78), 56.3% were men, 19.7% required invasive mechanical ventilation (IMV), and 44.8% died during hospitalization. Potential predictors were selected based on a thorough literature review. Generalized Additive Models were used to assess outcomes and predictors, while LASSO regression was employed to develop the mortality score. Risk groups stratification were divided based on the predicted death probability as intermediate, high, and very high risk.

Calibration plot of the AB2CO risk score

**Figure:**
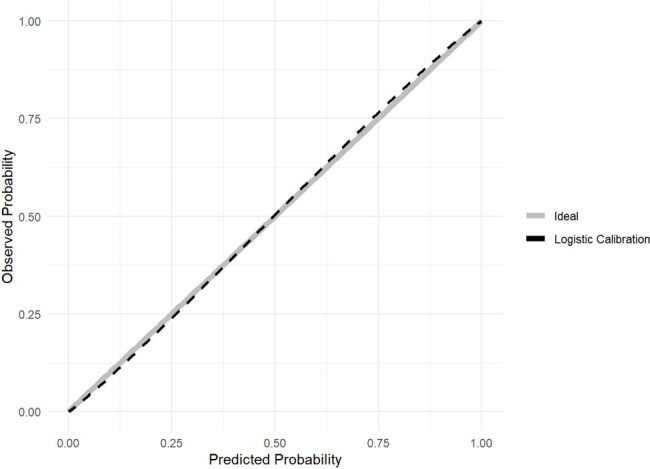

**Results:**

The final model, named AB_2_CO, comprised 6 variables: age, pO_2_/FiO_2_, respiratory function (respiratory rate or indication of IMV), COPD, and obesity. The AB_2_CO model showed an area under the Receiver Operating Characteristic curve of 0.781 (95% CI 0.744 to 0.819), good overall performance and an excellent calibration. Furthermore, on a complete case analysis the score achieved a discrimination ability of 0.783 (CI 95% 0.743-0.822), and it was compared with other existing scores and exhibited better performance than all of them.

**Figure:**
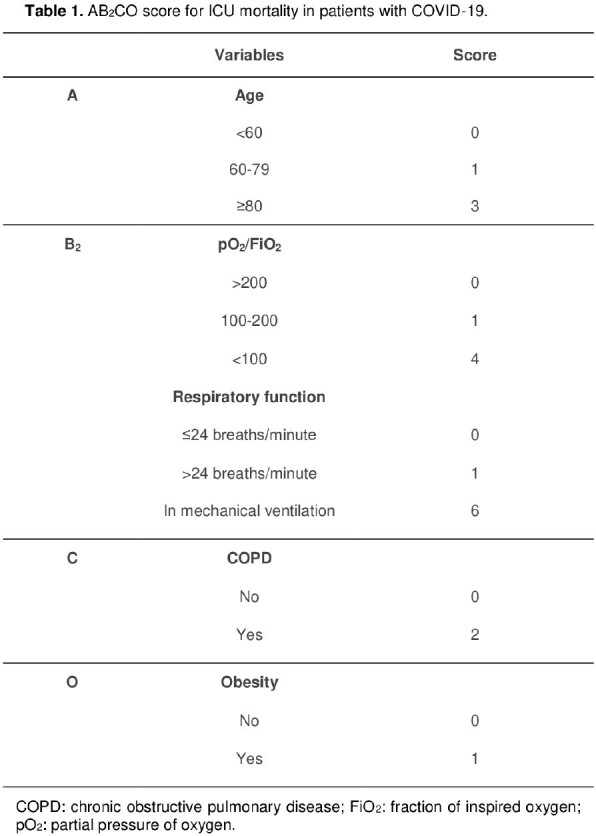

**Conclusion:**

The AB_2_CO score is a simple and easy tool, based on only six varibles routinely available in ICU. It may provide guidance for clinical decisions.

Discrimination ability for each risk score applied in the Brazilian database of COVID-19 patients admitted to the intensive care unit, and comparison of the derived and other existing scores.

**Figure:**
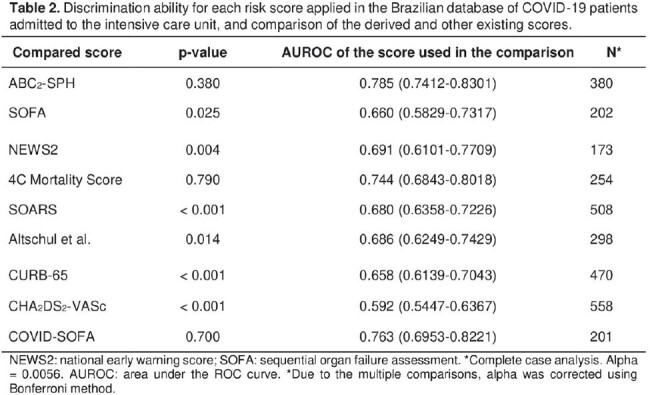

**Disclosures:**

All Authors: No reported disclosures

